# Etiology: Direct vs. Indirect Theories

**Published:** 1917-01

**Authors:** 


**A Monthly Review of Medicine and Surgery**

EDITOR AND PUBLISHER, DR. A. L. BENEDICT, 228 Summer St., corner of Elmwood Ave., Buffalo, (Address for all communications. Please make personal and telephone calls before 1 P. M.)

Third, in age, on the western continent. Independent, but supports professional organizations. News limited mainly to Western N. Y. and Penn.

Subscription $2.00 a year; $3.00 for two years in advance. Changes and errors in address or failure or duplication of delivery, should be reported immediately.

Yearly Volume 72 JANUARY, 1917 Number 6

### Etiology: Direct vs. Indirect Theories.

About the time of the Spanish-American and Boer Wars, the fly theory of the transmission of typhoid was in its ascendancy, some going so far as to consider it the principal means of communication. Before this, communication by ice was emphasized, certain extremists minimizing the influence of liquid ice. At various other times, personal contact and milk conveyance have been demonstrated beyond doubt, yet, at present, as years ago, it is almost universally conceded that the paramount factor in the spread of typhoid is drinking water, contaminated pretty directly by excrement.

Certain diseases, as yellow fever, malaria and sleeping sickness, have been shown to be conveyed by insect bites. Even with an abundant potential supply of the ultimate germs, the conveyance of these diseases is, at least, phenomenally rare without the presence of the particular intermediate host, and at most, possible only by a close imitation of the natural method of conveyance. These facts have quite naturally suggested that all diseases may be similarly transmitted, so that prophylaxis might be reduced nearly to a study of entomology and a safeguarding of the insect from the original host, or of the ultimate human host from the insect. But actual experience, positive as well as negative, has shown that the insect transmission applies only to a comparatively few diseases.

At one time, the idea that pulmonary and other respiratory tuberculous lesions were due to direct inhalation of bacilli from dust, seemed too simple to be accepted by the scientific practitioner. It was held that the infection was always haematogenic—as undoubtedly it may be, for the lungs as for other tissues—that the incubation period was not merely longer than for many other infections, but that it dated back to childhood or infancy, or was actually of foetal origin. More or less associated with these conceptions was that tuberculosis of all forms entered through the alimentary canal, that it was invariably due to infected milk—and then came Koch's surprising denial of this method in toto. We need not discuss the now well-known discrimination of human and bovine types in the human, nor the controversy as to the elimination of tubercle bacilli in the faeces, nor the qualifications requisite in adopting any one theory as to transmission. The main fact to be considered is that the profession has returned to an acceptance of the inhalation theory to account for the majority of respiratory localizations of tuberculosis. Indeed, from the practical standpoint of individual prophylaxis and treatment, we have gone a long way back toward the old conception of tuberculosis as a constitutional disease.

The public drinking cup and towel have nearly disappeared and one can even get closet seat covers from a slot machine, and these advances toward cleanliness and decency undoubtedly reduce the incidence of disease, yet the individual physician, dealing with the individual case, is justly sceptic as to the probability of these routes of infection for venereal or other infectious diseases, and in spite of the large and interesting literature on syphilis insontium, the actual prevailing opinion of the profession is that this and other venereal diseases are, in the great majority of instances, due to the cause ascribed to them several centuries ago.

For several years, almost every infection of unknown germ was ascribed to a filtrable, ultramicroscopic virus. But controls have shown that even a porcelain filter may not be impervious to bacteria and other germs of ordinary size; that the putative ultramicroscopic bacterium of syphilis is, in all probability not a bacterium at all and, at any rate, that it is one of the largest disease germs known, up to the group commonly termed parasites. In other instances hypothetic ultramicroscopic germs have been isolated and, while somewhat smaller than the average, by no means justify this term. In fact, the conception of an extremely minute underworld of life, has nothing to substantiate it.

The fad of ascribing various infections to bad teeth and tonsils is still with us. Far be it from us to advocate a dental cavity, or a tonsilar crypt teeming with bacteria, as conducive to health, but the crude fact is that the infections, rheumatism, pneumonia, tuberculosis, et al., commonly ascribed to these reservoirs of germs often occur in those whose teeth and tonsils are in remarkably good condition, and often fail to occur in those apparently most fitted to develop them. The present European war will be notable in medical history for many things; among them for the first fairly wide-spread acceptance of the anti-bacterial power of saliva. Ever since the discovery of bacteria as causes of infection, the profession has wondered at the escape of ignorant human beings, dogs and other animals, from the logical result of licking wounds, and has ascribed it to "resistance." Now we have a scientific endorsement of a method of therapy that antedates the human race.

Very recently, the conception of pyorrhea as a specific process has culminated in the announcement of the amoeba and an equally specific means of its destruction. That an amoeba is usually present in pyorrhea is admitted, but it is not definitely established whether it is always present in pyorrhea, and it is pretty well demonstrated that it may be present without distinct evidence of pyorrhea. Sceptic reports are beginning to appear concerning the lethal action of emetine, and the real etiologic significance of the amoeba.

There is no obvious reason for presupposing a specific germ for the seperation of the gum from the hair line between enamel and unprotected dentine, nor for the caries that might be expected to follow from ordinary saprophytes. Certain stomatologists have included under the term pyorrhea, cases of recession of the gums and dental caries, without pyorrhoea in the literal sense at all. We may be pardoned for mentioning a case observed at intervals for some years with a dentist who switched from the acidity to the amoebic theory, but persisted in regarding the recession of the gums as justifying the term pyorrhea, although there was never a discharge of pus from about the roots of the teeth. Our theory was that ordinary senile changes were responsible for the recession of the gums and that such small cavities as developed in the exposed roots were inevitable when one considers the ubiquity of bacteria capable of producing caries wherever the enamel is lacking. The lack of literal pyorrhoea was ascribed to the patient's zeal in using thread and tooth brush. The patient sustained a fracture of the jaw, was unable to use thread between the teeth and was compelled to use the brush very gently. The result was that the recession of the gums disappeared entirely.

In discussing this subject from the conservative—possibly some critic may say the reactionary—standpoint, we do not wish to give the impression that established facts in etiology are doubted, or that what appears to be the most direct method of infection is always the real route followed. A priori conceptions must always yield to demonstration of facts.

But, in general, the natural, logical theory, corroborated by observation through a long period of years should be adhered to until definite evidence is submitted for discarding it.

